# A Rare Case of Advanced-Stage Small Cell Carcinoma of the Ovary Identified During the Third Trimester of Pregnancy

**DOI:** 10.7759/cureus.55696

**Published:** 2024-03-06

**Authors:** Lua Saylany, Hasthika Ellepola, Elisha Broom

**Affiliations:** 1 Obstetrics and Gynecology, Logan Hospital, Logan, AUS; 2 Maternal-Fetal Medicine, Logan Hospital, Logan, AUS

**Keywords:** preterm delivery, ovarian cancers, pregnancy, cancer in young, small cell carcinoma of the ovary

## Abstract

Small cell carcinoma of the ovary, hypercalcemic type (SCCOHT) is a rare form of aggressive ovarian malignancy linked with mutations in the SMARCA4 gene. This disease predominantly affects young women within the first five decades of life and is associated with poor overall long-term survival, particularly when diagnosed in the advanced stage of the disease. Due to the low incidence of the condition and limited literature, current clinical decision-making is based on a small number of case series and case reports. We present an extremely rare case of SCCOHT diagnosed in a young female during her third trimester of pregnancy, requiring preterm delivery via cesarean section with simultaneous unilateral oophorectomy and salpingectomy.

## Introduction

Primary small cell carcinoma of the ovary, hypercalcemic type (SCCOHT) is highly aggressive and historically has a poor prognosis. The majority of women diagnosed with SCCOHT are women who are less than 50 years old [1]. The likelihood of survival is strongly associated with the stage of the disease at the time of diagnosis rather than the treatment modalities used [2,3]. The limited data available describes a one-year survival rate of only 50%, with an overall five-year survival rate of approximately 10% [3]. There is no standard, evidence-based treatment approach among the limited reported cases. However, the majority of multidisciplinary teams across various healthcare institutions around the world approach the management of SCCOHT with a combination of surgery and platinum-based chemotherapy. Surgical management often involves either unilateral salpingo-oophorectomy (fertility-sparing) or total abdominal hysterectomy with bilateral salpingo-oophorectomy, depending on the patient's age, preference, and stage of disease at the time of diagnosis. There is also a proportion of patients who undergo widespread abdominal radiotherapy. Unfortunately, these aggressive approaches lead patients to incur significant morbidity from the adjuvant therapy without offering long-term survival benefits [1-4].

The development of ovarian cancer in pregnancy is a rare occurrence [5,6]. One literature review states that ovarian cancer is diagnosed during pregnancy in approximately one in 15,000 to one in 32,000 pregnancies [7]. Furthermore, there are scarce reports of antenatal diagnoses of SCCOHT, in particular, and therefore there is limited data regarding evidence-based management and overall outcomes. We present a case of advanced-stage SCCOHT diagnosed in the third trimester, warranting preterm delivery and surgical intervention.

## Case presentation

A 27-year-old female G2P1 at 32 weeks gestation presented to the maternity assessment unit at a hospital with two weeks of new left upper quadrant pain and associated non-specific symptoms of nausea, vomiting, loose bowel motions, and mild intermittent dyspnea. She reported normal fetal movements and had no vaginal bleeding or fluid loss. She previously experienced one uncomplicated vaginal birth at term and had no significant medical conditions. She was not taking any medications and had no history of smoking, alcohol use, or illicit drug use. She had no known family history of ovarian, breast, or uterine malignancy. On examination, she had a large palpable firm abdominal mass in the left upper quadrant extending to the epigastric region, displacing the uterus to the far right of her abdomen. Fetal cardiotocography was within normal limits.

Urgent transvaginal and abdominal ultrasounds were performed, demonstrating a large heterogeneous solid-appearing structure with internal vascularity superior to the uterus measuring 22.3 x 17.7 x 12.6 cm. The ovaries were not visualized and there was approximately 2 liters of intraperitoneal fluid identified. Pathology results showed that her CA 125 level was 944 U/mL (normal range: <35.0 U/mL), lactate dehydrogenase was 1120 U/L (normal range: <234 U/L), C-reactive protein was 170 mg/L (normal range: <5 mg/L), and her serum calcium was within normal limits.

**Figure 1:**
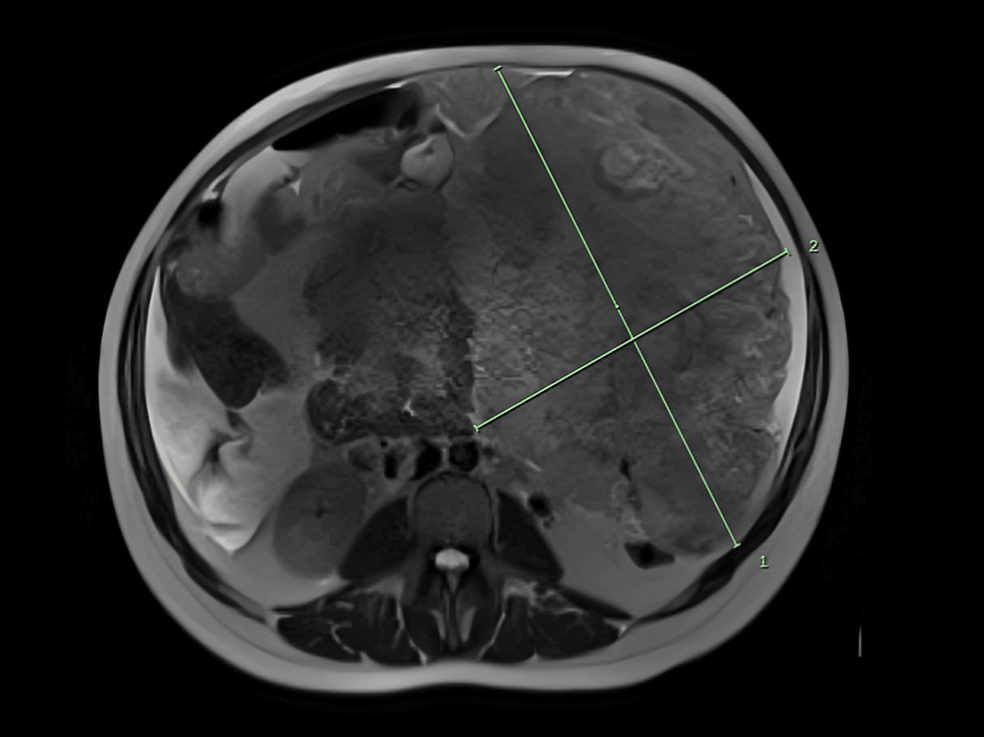
MRI T2 axial plane demonstrating a complex left upper abdominal mass with a maximum diameter of 26 cm.

**Figure 2:**
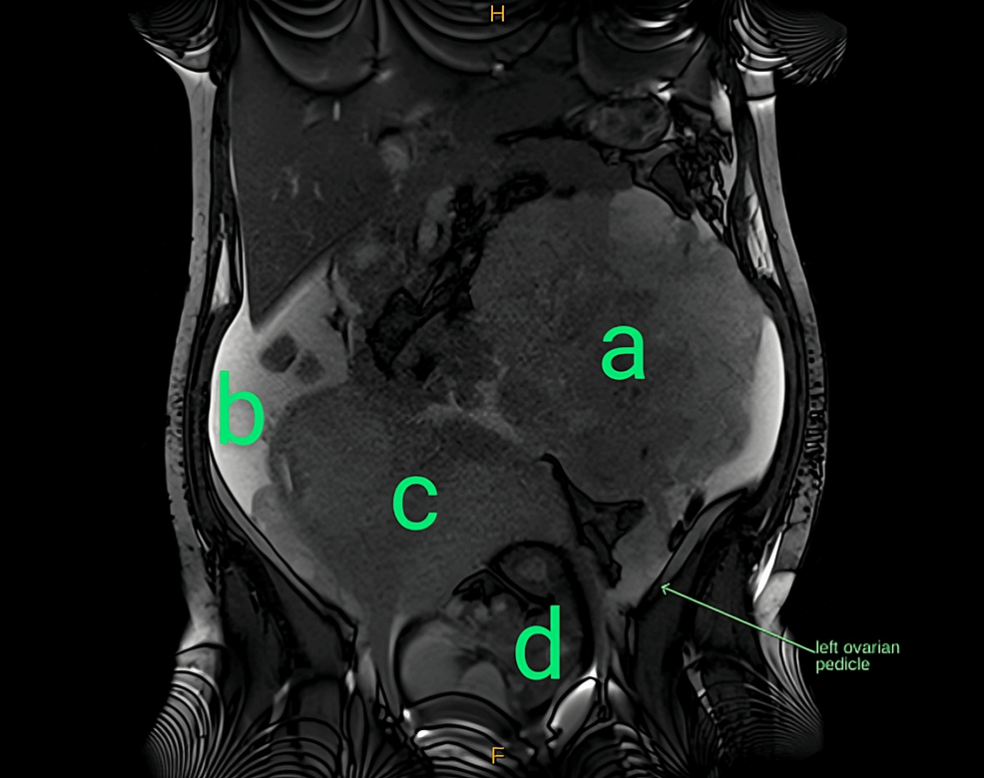
MRI T2 coronal plane showing the left ovarian pedicle (arrow) with insertion into the complex left upper abdominal mass (a), consistent with an ovarian origin; moderate ascites can also be appreciated (b). Uterine displacement (c) and the preterm fetus are also shown (d).

The patient was immediately transferred to a tertiary-level center for abdominal and pelvic magnetic resonance imaging (MRI) and surgical planning. MRI demonstrated a 26-cm left-sided abdominal mass with the left gonadal vein and ovarian pedicle traced into the mass with no residual normal left ovary identified​. Additionally, there were suspicious left para-aortic lymph nodes up to the level of the left renal vein, moderate ascites, and small bilateral pleural effusions identified. No bony abnormalities or omental caking were appreciated (Figures 1, 2).

In the context of elevated tumor marker CA 125 and the MRI findings, a late-stage primary ovarian malignancy was suspected. Therefore, in discussion with the patient and her family, elective surgical management was scheduled for one week later when the patient would be at 33 weeks gestation, performed by gynecological oncology specialists in collaboration with obstetric specialists. At that time, the patient opted against more aggressive surgery, prioritizing a quicker recovery for her newborn and choosing to await histopathology results. She underwent a cesarean section, left salpingo-oophorectomy, and omental biopsies. Histopathological evaluation of the tissue samples demonstrated a high-grade BRG1-deficient malignancy consistent with SCCOHT, with fallopian tube and omental involvement confirmed. She was diagnosed with FIGO (International Federation of Gynecology and Obstetrics) stage III malignancy, for which genetic counseling and palliative chemotherapy were recommended. She accepted these recommendations and is currently undergoing this follow-up management. The baby spent some time in special care following delivery, experiencing no significant complications beyond those typically expected for the gestational age at the time of birth.

## Discussion

SCCOHT is a rare malignancy that predominantly arises in young females and is associated with a poor prognosis. A review of the literature demonstrates only a few cases of SCCOHT in pregnancy [2,8]. The clinical presentation of SCCOHT is typically non-specific with the most frequently mentioned symptoms being localized abdominal pain and abdominal distension [2], which are symptoms easily masked in pregnancy. Definitive preoperative diagnosis of SCCOHT is generally challenging without a tissue biopsy [2,9], especially when compounded by pregnancy. Histopathological examination remains the gold standard for diagnosis of SCCOHT [1,2,9]. In this case report, suspected ovarian malignancy was identified in a pregnant female at 27 years old, who had no known significant medical history or risk factors. A multidisciplinary decision was made, in consultation with the patient, to expedite delivery of the fetus and to urgently commence multi-modal management of the suspected malignancy, later confirmed histologically to be SCCOHT.

Early diagnosis and treatment of SCCOHT have been found throughout the literature to be the most crucial predictor of long-term survival. A key challenge in early diagnosis of patients with SCCOHT is the nonspecific nature of the clinical presentation. Although it has been reported that up to two-thirds of patients with SCCOHT have hypercalcemia demonstrated in their pathology, less than 10% of these patients experience symptoms of hypercalcemia [1,2]. The literature also describes the role of CA 125 in distinguishing between benign and malignant ovarian masses. Serum CA 125 is elevated in 50-75% of patients with the diagnosis of SCCOHT [2,9]. Nonetheless, caution should be used when interpreting tumor markers in the diagnosis of ovarian malignancy during pregnancy as it can be normal during pregnancy to have elevated maternal serum tumor marker levels, particularly CA 125 [10]. Additionally, the primary imaging modality used to evaluate suspected ovarian malignancy is transvaginal pelvic ultrasound. Computed tomography (CT) and/or MRI are subsequently used not only to enhance the characterization of suspected malignancies but also to gather information for staging purposes. CT, being more accessible, is more frequently utilized compared to MRI. However, in pregnant patients, MRI is preferred due to its avoidance of high-contrast resolution and radiation exposure to the fetus in comparison to CT [11]. In the presented case, the patient did not have hypercalcemia on her pathology tests; however, notable findings included elevated CA 125 levels and the presence of a prominent likely ovarian mass with ascites appreciated on both ultrasound and MRI.

Recent literature has described the inactivation of the SMARCA4 gene, which encodes the BRG1 protein, as a key contributor to the development of SCCOHT, and has been identified in upward of 95% of patients with a diagnosis of SCCOHT [12,13]. In the presented case report, the tissue biopsies were highly deficient in the BRG1 protein, which further supports the final diagnosis of SCCOHT. Increasing data demonstrate that assessing for the SMARCA4 mutation/BRG1 protein is a useful diagnostic marker for SCCOHT [14] and provides possible avenues for implementing targeted treatment options. A particular area of importance, requiring further research, is genetic counseling and screening in patients and at-risk family members. Some studies have discussed the potential role of risk-reducing bilateral salpingo-oophorectomy in germline SMARCA4 mutation carriers [15,16]; however, this nascent idea is not currently substantiated in the literature and is a clinical challenge given the early age of disease onset. Limited studies have reported offering risk-reducing bilateral salpingo-oophorectomy in highly selective cases, such as siblings in an affected family [16]. In the presented case, this is an area of further follow-up by the genetic counseling clinicians for the patient’s biological children and close relatives.

Due to the absence of any prospective studies on the topic of SCCOHT, management recommendations are based on limited case series, leading to varied treatment approaches. The typical treatment strategy includes radical surgery, followed by adjuvant chemotherapy, and, if deemed necessary, radiotherapy [2,3,16]. The ideal surgical approach is not clear, with some studies suggesting aggressive debulking surgery with bilateral salpingo-oophorectomy and/or hysterectomy [2,15] while other studies suggest a role for more conservative, fertility-conserving therapy (i.e., unilateral salpingo-oophorectomy) as a preferable approach, particularly in early stages of the disease [1,3]. Adjuvant high-dose chemotherapy treatment is generally recommended for all patients diagnosed with SCCOHT and has particularly been important in the context of fertility preservation [17,18]. The value of radiotherapy is not clearly established in the literature, with some studies citing improved outcomes [1-3,18] and others demonstrating inconclusive outcomes [2,19], with most studies on the topic limited by small patient cohorts. It is clear that an individualized approach is required, primarily considering the stage of the disease, which is highly indicative of the prognosis. The patient’s quality of life and morbidity in the context of recommended treatments should then be considered with clear communication of likely prognosis and the scope of benefits and harm that can come with intervention options. In the presented case, late-stage malignancy was suspected in the third trimester of pregnancy, hence preterm delivery and surgical intervention with unilateral oophorectomy and salpingectomy were carried out. Upon confirmation of late-stage disease, palliative chemotherapy was recommended and accepted by the patient.

## Conclusions

The case outlined describes a pregnant woman in her third trimester who exhibits clinical features highly indicative of advanced-stage ovarian cancer, prompting the need for premature delivery alongside immediate surgical intervention, followed by palliative chemotherapy. Subsequent histopathological analysis confirmed a diagnosis of SCCOHT. Unfortunately, SCCOHT is not only a rare form of ovarian cancer, but it also carries a poor prognosis, particularly when diagnosed in the late stage of the disease. The management typically involves a multimodal approach; however, the extent of surgical intervention and the intensity of adjuvant therapies are subject to debate in the literature. Further international consensus is required to establish a standard protocol. The relatively recent discovery of the association between SMARCA4 mutations and SCCOHT opens a potential pathway for more effective prevention and, potentially, treatment targeting the driver mutation. Establishing a case registry would help identify treatment variations to benefit future patients diagnosed with SCCOHT.
